# Bringing p53 Back: A Prion-Powered Attack on Retinoblastoma

**DOI:** 10.7150/ijbs.113116

**Published:** 2025-09-22

**Authors:** Yuyan Ma, Siqi Yan, Weiming You, Peili Wang, Wangxiao He, Yu Yao, Xiaoqiang Zheng

**Affiliations:** 1 Department of Medical Oncology, The First Affiliated Hospital of Xi'an Jiaotong University, Xi'an 710061, China.; 2 Department of Hepatology, The Second Affiliated Hospital of Xi'an Jiaotong University, Xi'an 710004, China.; 3 Institute for Stem Cell & Regenerative Medicine, The Second Affiliated Hospital of Xi'an Jiaotong University, Xi'an 710004, China.; 4 National & Local Joint Engineering Research Center of Biodiagnosis and Biotherapy, The Second Affiliated Hospital of Xi'an Jiaotong University, Xi'an 710004, China.

**Keywords:** Retinoblastoma, HDMX, p53, peptide, prion-like self-assembly

## Abstract

Retinoblastoma (RB) represents the most common primary intraocular malignancy in children, driving a critical need for innovative, targeted therapies that enhance tumor control while preserving vision. Current chemotherapy regimens, such as melphalan, can result in significant systemic toxicity and ocular side effects, underscoring the urgency for safer, more selective treatments. Here, we comprehensively report the design and evaluation of a prion-like self-assembling peptide prodrug (Pri-MP) that exploits the elevated macropinocytic uptake in RB cells to deliver an HDMX-targeting peptide, thereby restoring p53 function. Using single-cell RNA sequencing, we identified a key role for Rac1-PAK1 signaling in driving RB-specific macropinocytosis, which facilitated selective intracellular accumulation of Pri-MP through Au(I)-mediated reversible assembly. This strategy enabled potent p53-dependent apoptosis, prompting marked cell cycle arrest and robust tumor suppression *in vitro*. In an orthotopic mouse model, intravitreal Pri-MP significantly curtailed tumor burden and demonstrated the potential for enhanced antitumor activity when combined with melphalan, without imposing systemic toxicity or injuring healthy ocular structures. Mechanistically, Pri-MP antagonizes HDMX, lifting its inhibition of p53 and triggering pro-apoptotic transcriptional programs. By leveraging prion-inspired delivery to achieve high specificity and enhanced safety, this approach addresses a longstanding challenge in RB therapy, where efficient tumor targeting remains paramount and vision preservation is essential. Our *in vivo* findings further confirm the transformative potential of this platform for tumor-specific p53 reactivation, potentially applicable to other neuroectodermal malignancies. Pri-MP thus holds promise as a next-generation modality for eye-preserving RB treatment, meriting further investigation in clinical settings to advance safer, more effective management of this devastating pediatric cancer.

## Introduction

Retinoblastoma (RB) is the most common intraocular malignancy in children, posing a significant global health burden 1, 2. For advanced cases, enucleation remains a curative approach, but preserving vision is a primary goal for patients with localized disease 3. Current treatment regimens rely heavily on intra-arterial and intravitreal chemotherapy, primarily using melphalan 4. However, systemic toxicity, ocular side effects, and the potential for chemoresistance limit the long-term success of these treatments 5, 6. This highlights an urgent need for novel therapeutic strategies that can efficiently, rapidly, and safely eliminate intraocular tumors while preserving ocular function 7, 8.

The emergence of targeted therapies has provided new opportunities to address these challenges 9, 10. Unlike conventional chemotherapy, targeted drugs can exploit tumor-specific molecular vulnerabilities to achieve high therapeutic efficacy with minimal systemic toxicity 11, 12. Genetically, RB is characterized by RB1 inactivation, which leads to the upregulation of MDMX, an oncogenic inhibitor of p53 13, 14. Notably, most RB tumors retain wild-type TP53, making them uniquely sensitive to therapeutic strategies that restore p53 function 15, 16. However, prior attempts to inhibit HDMX (or its homolog HDM2) systemically have led to severe toxicities, limiting clinical translation 17, 18. Therefore, an ideal treatment strategy would selectively antagonize HDMX in RB cells, reactivate p53, and induce tumor regression without significant off-target effects.

RB cells exhibit a remarkable propensity for macropinocytosis, an endocytic process that enables them to engulf extracellular components to support their high metabolic demands 9, 11. This feature sets them apart from normal retinal cells and presents an opportunity for selective drug delivery 19. Notably, prion proteins, known for their neurotropism, naturally exploit macropinocytosis for targeted uptake in neural tissues 16, 18. Compared to normal neurons, RB cells demonstrate even higher macropinocytic activity, making them particularly susceptible to prion-inspired nanostructures 8, 14. This cellular behavior suggests that prion-bionic delivery systems could provide a powerful means of tumor-selective drug targeting while sparing healthy tissues 20.

Building upon this rationale, we developed Pri-MP, a prion-like nanostructure engineered from an HDMX-targeting peptide (MP) 21-23. By leveraging thiol modification and Au(I)-mediated reversible self-assembly 24-26, Pri-MP forms stable nanostructures that selectively undergo macropinocytic uptake in RB cells 27. This strategy enables efficient intracellular delivery, leading to robust p53 activation, cell cycle arrest, and apoptosis. *In vivo*, studies demonstrate that Pri-MP significantly suppresses RB tumor growth while exhibiting excellent compatibility with melphalan chemotherapy. Importantly, Pri-MP overcomes the toxicity barriers associated with prior p53-targeting strategies, maintaining a favorable safety profile. Our findings establish a biomimetic approach for RB treatment by harnessing prion-inspired nanotechnology for selective p53 reactivation. This method not only provides a highly tumor-specific and low-toxicity therapeutic strategy for RB but also offers a broader platform for using prion-like nanoparticles in targeted cancer therapy. Given its translational potential, Pri-MP represents a promising next-generation therapeutic modality, warranting further clinical investigation for RB and other neuroectodermal malignancies.

## Results

### Macropinocytic properties in retinoblastoma cells

**Figure 1:**
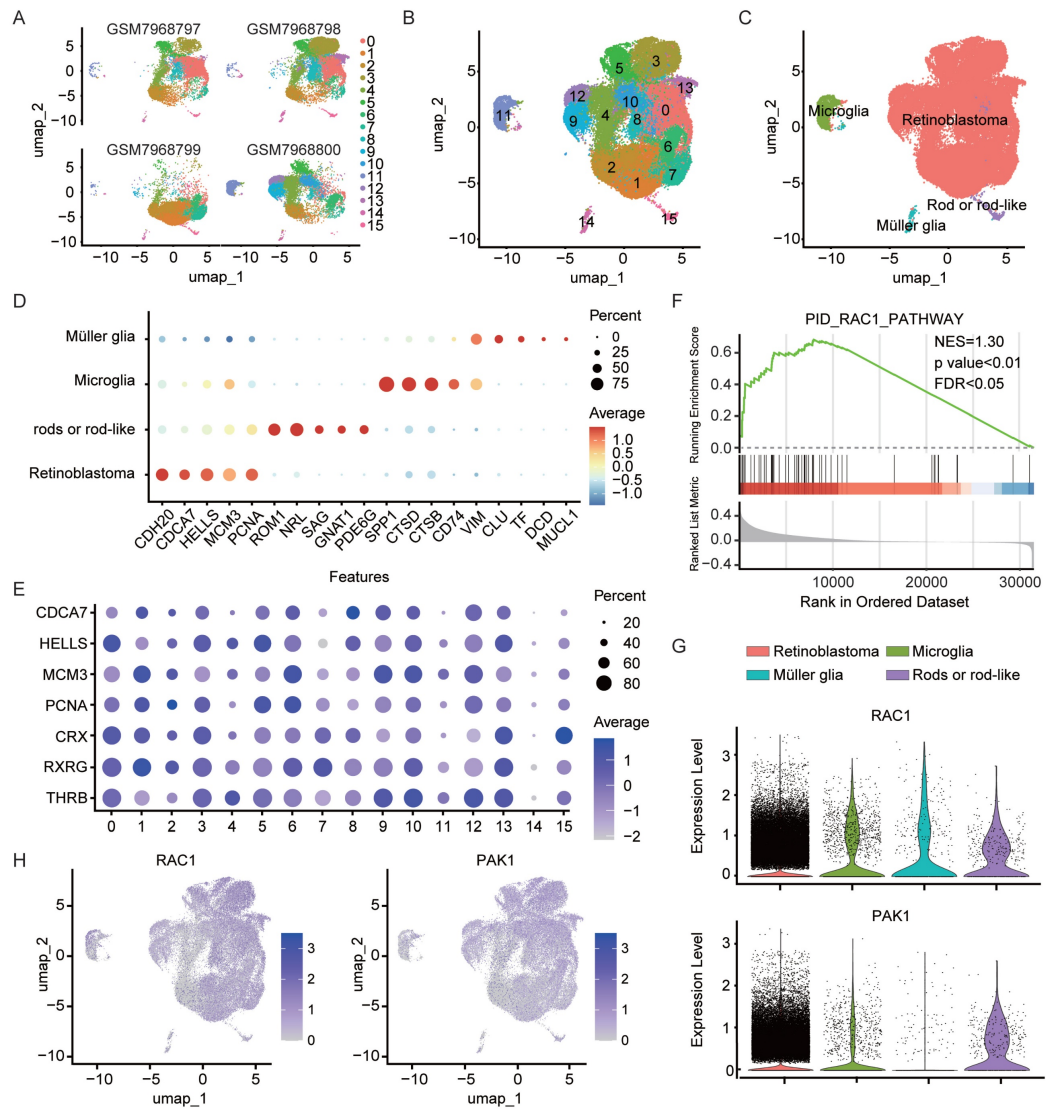
Single-cell analysis reveals enhanced macropinocytic activity in RB cells versus normal intraocular cells. (A-B) UMAP projection of single-cell transcriptomes from four independent retinal tissue samples(A) and integrated UMAP visualization(B) of the combined dataset (GSE249995) are presented, with individual points representing cells and colors indicating computationally annotated clusters. (C) Cell-type annotation was achieved using published single-cell RNA-seq datasets through reference-based mapping. (D) Specific gene markers were identified for each cell type. (E) Expression levels of marker genes associated with the retinoblastoma cell class are shown. (F) Gene set enrichment analysis (GSEA) of genes in the Rac1 pathway (NES=1.30). (G) A violin plot illustrates the expression levels of Rac1 and PAK1 across different cell types. (H) The t-SNE visualization depicts Rac1 and PAK1 expression patterns, with gradient colors representing normalized expression levels.

In this study, we leveraged single-cell RNA sequencing data (GSE249995) containing human retinoblastoma (RB) samples from the Gene Expression Omnibus (GEO) database. A total of 54,420 cells were obtained from the intraocular tissues of four RB patients following stringent quality control. Principal component analysis (PCA) was first applied for dimensionality reduction, after which UMAP and clustering methods were used to partition the high-quality cells into 16 clusters (Fig. 1A). Cross-sample integration analysis showed a high degree of consistency in the distribution of cell subpopulations across different patients (Fig. 1B). To minimize the impact of cell state variability on clustering, we first regressed out cell cycle effects (Fig. S1A). Next, copy number variation (CNV) was inferred using inferCNV with publicly available non-neoplastic retinal cells (GSE196235) as reference. Several clusters exhibited pronounced CNV signals (Fig. S1B) and enrichment of tumor-associated transcriptional features, and were thus classified as retinoblastoma. Negative selection analysis further confirmed their malignant origin, as no canonical immune or stromal markers were detected in these clusters (Fig. S1C) 28, with their respective marker expression patterns displayed in Fig. 1D and 1E. Notably, RB cells constituted the largest proportion among all clusters, suggesting a dominant role for these cells in the tumor microenvironment. Further gene set enrichment analysis (GSEA) demonstrated significant enrichment of the Rac1 signaling pathway in RB cells (Fig. 1F, p < 0.01), accompanied by elevated expression of Rac1 and its downstream effector molecule p21-activated protein kinase 1 (PAK1) (Fig. 1G, p < 0.05). Previous studies have revealed that macropinocytosis is dependent on actin polymerization and Rac1/PAK1 signaling 25, 29. Therefore, we postulated that RB cells acquire macropinocytic properties by activating this pathway, thereby enhancing the uptake of metabolic nutrients. Further annotation of Rac1 and PAK1 expression, both key regulators in macropinocytosis, revealed higher levels of these molecules in RB tumor tissues compared with normal retinal surrounding cells (Fig. 1H), providing a molecular basis for the heightened macropinocytic activity of RB cells.

### Development of a prion-like self-assembling peptide prodrug (Pri-MP)

**Figure 2:**
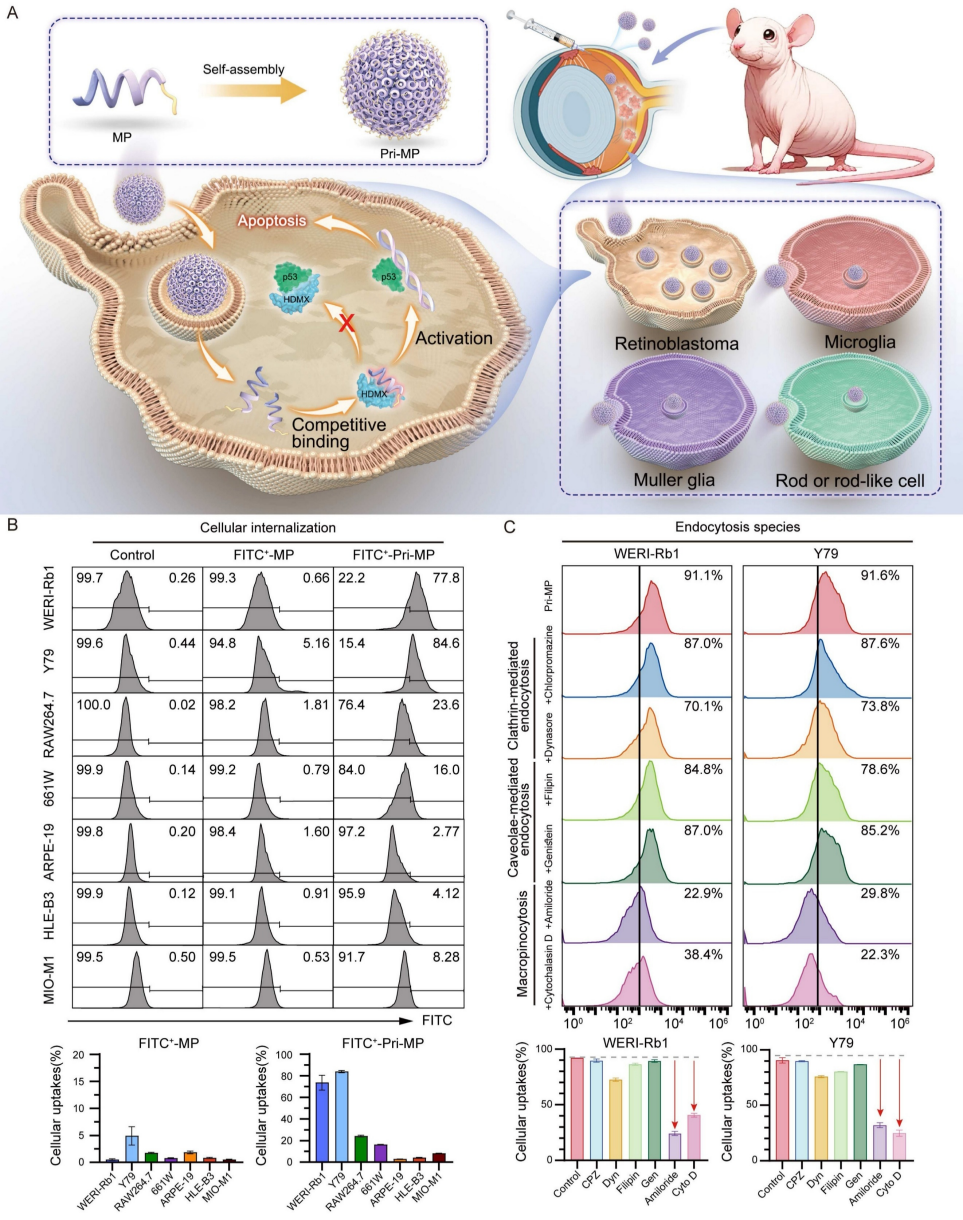
Schematic illustration of the prodrug Pri-MP design and its cellular internalization mechanism. (A) The MP polypeptide, with a high affinity for HDMX, self-assembles into the prodrug Pri-MP in one step. Pri-MP is effectively internalized by RB cells through macropinocytosis, exerting antitumor effects by blocking the p53-HDMX interaction, thus inhibiting HDMX's negative regulation of p53. (B) The efficiency of cellular uptake of FITC-labeled Pri-MP and FITC-labeled MP was assessed using flow cytometry in retinoblastoma cell lines (WERI-Rb1 and Y79), various intraocular normal cell lines (including 661W, ARPE-19, HLE-B3, and MIO-M1), as well as the RAW264.7 cell line. (C) Flow cytometry analysis of Pri-MP cellular internalization mechanisms. Macropinocytosis inhibitors (amiloride and cytochalasin D) significantly suppress Pri-MP uptake in RB cells (WERI-Rb1 and Y79) compared to clathrin-mediated endocytosis inhibitors (chlorpromazine and dynasore) and caveolae-mediated endocytosis blockers (filipin and genistein).

Inspired by these findings, we designed a prion-like self-assembling peptide prodrug system (Pri-MP) that leverages macropinocytosis to achieve selective internalization into RB cells (Fig. 2A). We first employed the deep learning algorithm AlphaFold-Multimer to predict the binding conformation between the peptide and HDMX (Fig. S2A). From these analyses, we identified a candidate peptide MP with a binding free energy (ΔG = -12.8 kcal/mol) that is significantly lower than that of the p53/HDMX complex (ΔG = -10.7 kcal/mol) (Fig. S2B). Compared with PMI, a previously identified peptide from our group capable of antagonizing HDMX 1, 30, 31, MP exhibited even stronger affinity for HDMX (Fig. S2B). HDM2, a homolog of HDMX, functions as a key negative regulator of p53 stability in RB tumors. Disrupting the HDM2-p53 interaction plays an essential role in reactivating p53 signaling and inducing antitumor effects 32. To clarify whether the peptide also interferes with HDM2-p53 binding, we performed comparative binding free energy (ΔG) calculations, which showed that the peptide displays minimal affinity for HDM2 (ΔG = -11.8 kcal/mol) relative to p53 (ΔG = -11.7 kcal/mol), suggesting negligible competitive binding and no significant disruption of the HDM2-p53 complex (Fig S2B). We therefore hypothesized that MP targets the binding interface of p53 and HDMX, competitively occupying this critical site to effectively block the negative regulation of p53 by HDMX, thereby restoring the normal biological function of p53. Leveraging the neurotropic properties of the prion-like structure format of prion-like domains characterized by low-complexity intrinsically disordered regions, MP was co-assembled into prion-like prodrug nanoparticles, termed Pri-MP, through Au(I)-mediated self-assembly, as described in our previous studies 24, 33, 34.

We next characterized the physicochemical properties of Pri-MP. Fourier-transform infrared (FTIR) spectroscopy showed C=O and N-H absorption peaks at 1680 cm^-1^ and 3300 cm^-1^, respectively, along with a characteristic Au-S absorption peak at 2950 cm^-1^ (Fig. S3). In addition, the overall baseline of the ultraviolet absorption spectrum of the self-assembled microprotein Pri-MP was higher than that of Pri-C (Fig. S4), indicating successful self-assembly of Pri-MP. Dynamic light scattering (DLS) revealed that Pri-MP forms stable nanostructures (~26.16 nm in diameter, PDI = 0.159; Fig. S5). Transmission electron microscopy (TEM) further supported the DLS results (Fig. S6). Furthermore, consistent with previous studies 1, Pri-MP is capable of disassembling into nanoparticles approximately 5 nm in size when exposed to high concentrations of glutathione (GSH), suggesting its potential for intracellular disassembly and subsequent peptide drug release (Fig. S7). Collectively, these data show that Pri-MP self-assembles into engineered microprotein particles of relatively uniform size and stable chemical properties.

To confirm the internalization capacity of Pri-MP in different RB cell lines, we labeled MP and Pri-MP with FITC, then compared uptake between tumor cells and non-tumor cells (e.g., human retinal pigment epithelial cells, lens epithelial cells). Compared with mouse retinal ganglion cells (661W), human retinal pigment epithelial cells (ARPE-19), mouse monocytic macrophage (RAW 264.7), human lens epithelial cells (HLE-B3), and retinal Müller stem cells (MIO-M1), FITC-labeled Pri-MP showed significantly higher uptake in the WERI-Rb1 and Y79 cell lines (Fig. 2B). Notably, under equivalent drug concentrations, the uptake ratio of Pri-MP in Y79 cells was reached ~5‑fold relative to 661W cells. Additionally, Pri-MP exhibited relatively low uptake in RAW 264.7 mouse macrophages, suggesting its potential to evade phagocytosis by the reticuloendothelial system. Collectively, these results demonstrate that Pri-MP is taken up preferentially by RB cell lines, but not by normal ocular cell lines.

To clarify the mechanism by which RB cells internalize Pri-MP, we employed the clathrin-mediated endocytosis inhibitors chlorpromazine (CPZ) and dynasore (Dyn), the caveolae-mediated endocytosis inhibitors filipin and genistein (GEN), and the macropinocytosis inhibitors amiloride and cytochalasin D (Cyto D). Following a 1-hour pre-treatment with these inhibitors, the cells were incubated with FITC-labeled Pri-MP for 6 hours. Under these conditions, pretreatment with the macropinocytosis inhibitors reduced Pri‑MP uptake in RB cells by up to ~40% relative to vehicle controls. In contrast, chlorpromazine (CPZ), dynasore (Dyn), filipin, and genistein (GEN) each reduced uptake by <10% (Fig. S8). With increasing concentrations of amiloride and Cyto D, the inhibitory effect on Pri‑MP uptake was further enhanced, reaching 73.0% (amiloride) and 55.4% (Cyto D) in WERI‑Rb1 cells, and 64.5% (amiloride) and 72.4% (Cyto D) in Y79 cells. However, even with dose escalation of other endocytic inhibitors, the inhibition remained below 20% (Fig. 2C). These findings indicate that the uptake of Pri-MP by RB cells is primarily mediated by macropinocytosis.

### Pri-MP exerts antitumor effects *via* activation of the p53 pathway

**Figure 3:**
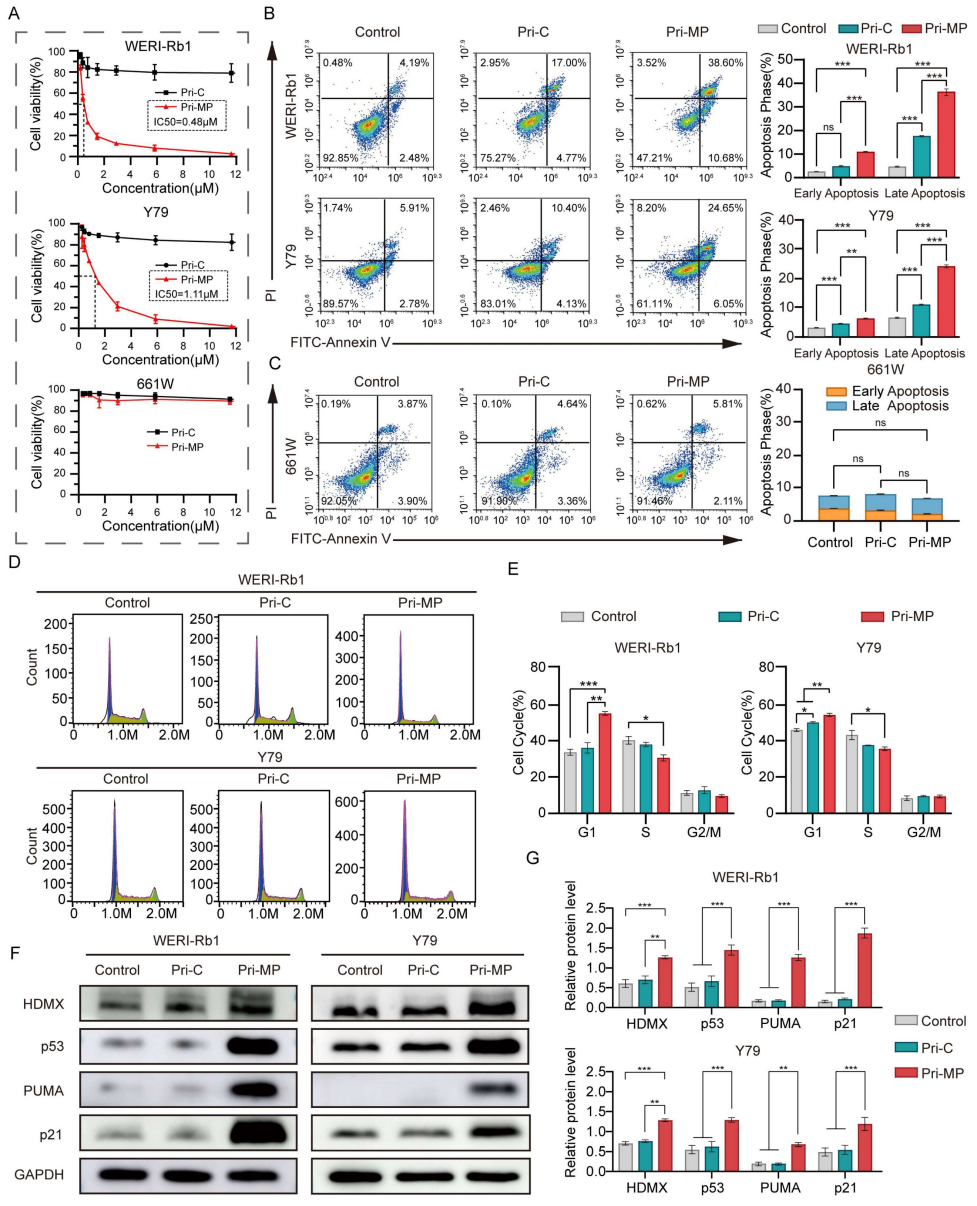
Pri-MP exerts antitumor effects via activation of the p53 pathway *in vitro*. (A) Dose-dependent growth inhibition of WERI-Rb1 and Y79 cells treated with Pri-MP or Pri-C, as determined by AlamarBlue assays (n=3). (B, C) Flow cytometric analysis reveals apoptosis induction in Pri-MP-treated WERI-Rb1/Y79 cells(B) and 661W (C) cells. Annexin V⁺/PI⁻ staining indicated early apoptotic cells, and Annexin V⁺/PI⁺ staining denoted late apoptotic cells. (D, E) The cell cycle distribution of RB cells following Pri-MP/Pri-C treatment for 24h was analyzed through PI staining and flow cytometry. (F, G) Western blot analysis (F) and quantification (G) of HDMX, p53, p21 and PUMA protein expression levels in WERI-Rb1 and Y79 cells (n=3). *, p < 0.05, **, p < 0.01, ***, p < 0.001, ns, not significant.

We subsequently assessed the antitumor efficacy of Pri-MP in RB cell lines WERI-Rb1 and Y79, both of which harbor wild-type TP53 and exhibit overexpression of HDMX. In cytotoxicity assays with a gradient of drug concentrations, Pri-MP exhibited a concentration-dependent cytotoxic effect on WERI-Rb1 cells, with an IC50 of 0.48 µM. In contrast, Pri-C displayed no concentration-dependent cytotoxicity, and even at higher concentrations, did not induce significant tumor cell killing. Similar results were observed in the Y79 cell line, with an IC50 of 1.11 µM (Fig. 3A). In mouse retinal ganglion cells (661W), Pri-MP did not elicit substantial cytotoxicity, which may be attributable to its tumor-selective uptake and relatively low uptake rate in normal retinal ganglion cells. Taken together, these data suggest that Pri-MP significantly inhibits RB cell proliferation without causing notable proliferation toxicity in normal retinal ganglion cell lines.

We further examined the pro-apoptotic activity of Pri-MP *via* flow cytometry. Through Annexin V-FITC/PI double staining, it was shown that after Pri-MP treatment, WERI-Rb1 cell apoptosis was 6.62-fold that of the Control group and 2.11-fold that of the Pri-C group. The pro-apoptotic effect in the Y79 cell line was 3.23-fold the level of the control group and 1.98-fold that of the Pri-C group (Fig. 3B). These findings underscore the strong pro-apoptotic effect of Pri-MP on tumor cells. Meanwhile, in 661W mouse retinal ganglion cells, the apoptotic rate induced by Pri-MP at equivalent concentrations did not significantly differ from that in the control or Pri-C groups (Fig. 3C). Overall, Pri-MP markedly inhibits proliferation and promotes apoptosis in RB cells, but lacks a similar effect in normal retinal ganglion cells (661W). We also performed flow cytometry to evaluate cell-cycle distribution in RB cells following Pri-MP treatment. As shown in Fig. 3D and 3E, compared with the Control groups, Pri-MP treatment increased the proportion of WERI-Rb1 cells in the G1 phase by 63.7% and decreased S-phase cells by 24.1%. A similar trend was observed in Y79 cells. These results indicate that Pri-MP induces G1/S checkpoint activation, preventing entry into S phase, thereby delaying or arresting cell-cycle progression.

To test whether Pri-MP's antitumor activity is mediated by antagonizing HDMX and reactivating the p53 pathway, we performed Western blot analyses (Fig. 3F, 3G, and Fig. S9). In WERI-Rb1 cells, Pri-MP increased HDMX and p53 approximately 2.1-fold and 2.9-fold, respectively, relative to control, with HDM2 showing a similar increase. Y79 cells exhibited concordant changes, with a marked rise in p53. To assess the functional consequences of p53 elevation, we examined downstream effectors: in WERI‑Rb1, PUMA increased 8.5‑fold (Fig. 3F, 3G), Noxa rose accordingly (Fig. S9A, S9B), and the cell-cycle inhibitor p21 was also elevated (Fig. 3F, 3G). Pri‑MP-mediated activation of p53 downstream targets was likewise observed in Y79 cells. These data support a model in which Pri-MP disrupts HDMX-p53 binding, leading to accumulation of HDMX in an inactive state and thereby relieving HDMX-mediated repression of p53 transactivation. Functionally reactivated p53 then drives robust induction of the pro-apoptotic targets PUMA and Noxa and the cell-cycle regulator p21, consistent with apoptosis and G1/S checkpoint activation. In parallel, p53-dependent negative feedback accounts for the observed upregulation of MDM2, and stabilization within the MDM2-HDMX complex may contribute to increased HDMX levels.

To further elucidate the underlying mechanisms by which Pri-MP exerts its antitumor effects, we performed RNA-seq analysis of tumor cells following treatment. As shown in Figure S11, differentially expressed genes (DEGs) were identified using the criteria of |log₂FC| > 1 and a false discovery rate (FDR) < 0.05. The volcano plot revealed that Pri-MP treatment significantly upregulated 150 genes and downregulated 195 genes (Fig. S11A). A heatmap was generated to investigate the expression patterns of DEGs across samples further (Fig. S11B). The results demonstrated distinct clustering between the Pri-MP-treated and control groups, indicating substantial transcriptomic differences between the two conditions. To explore the biological pathways associated with the DEGs, we conducted Gene Set Enrichment Analysis (GSEA). The analysis revealed significant enrichment of the p53 signaling pathway in the Pri-MP-treated group, suggesting that Pri-MP may exert its inhibitory effects by activating p53-dependent stress response mechanisms (Fig. S11C). Moreover, the apoptosis pathway, a downstream effector of p53 signaling, was also significantly enriched (Fig. S11D), supporting the notion that Pri-MP may exert anti-tumor activity through activation of the p53 pathway and induction of apoptotic processes. Previous studies have reported that MDM2(HDM2) promotes RB cell proliferation through p53-independent regulation of MYCN translation 12. In the results of our analysis, Pri-MP treatment did not alter MYCN mRNA levels (Fig. S12), consistent with its selectivity for HDMX over HDM2. Our study enriches the related exploration of the HDM2/HDMX pathway in retinoblastoma.

To validate whether the anti-tumor effect of Pri-MP is p53-dependent, RNA interference was employed to knock down p53 expression. Quantitative polymerase chain reaction (qPCR) and Western blot analyses demonstrated successful inhibition of p53 at both mRNA (Fig. S13A) and protein levels (Fig. S13B) in WERI-Rb1 and Y79 cell lines. Subsequently, dose-dependent growth inhibition assays were performed with Pri-MP in si-p53-transfected WERI-Rb1 and Y79 cells (Fig. S13C). The inhibitory effect of Pri-MP in WERI-Rb1 and Y79 cells transfected with si-p53 has decreased. Furthermore, the apoptosis assay demonstrated that the pro-apoptotic effect of Pri-MP was significantly attenuated upon downregulation of p53 protein levels in RB cells (Fig. S13D, 13E). Western blot analysis revealed that compared with wild-type TP53 cell lines, the upregulation levels of downstream proteins p21 and Noxa induced by Pri-MP treatment were significantly attenuated in si-p53 WERI-Rb1 cells, with reduction rates of approximately 74.8% and 92.4%, respectively. A consistent trend was observed in Y79 cells, where the ability of Pri-MP to upregulate p21 and Noxa was diminished by approximately 75.8% and 72.6%, respectively, following p53 interference (Fig. S13F, 13G).

In summary, Pri-MP exerts its tumor-suppressive effects—namely inhibiting proliferation, arresting the cell cycle, and inducing apoptosis—through antagonizing HDMX and reactivating the p53 pathway.

### Pri-MP inhibits tumor growth in a mouse orthotopic RB model by activating the p53 pathway

**Figure 4:**
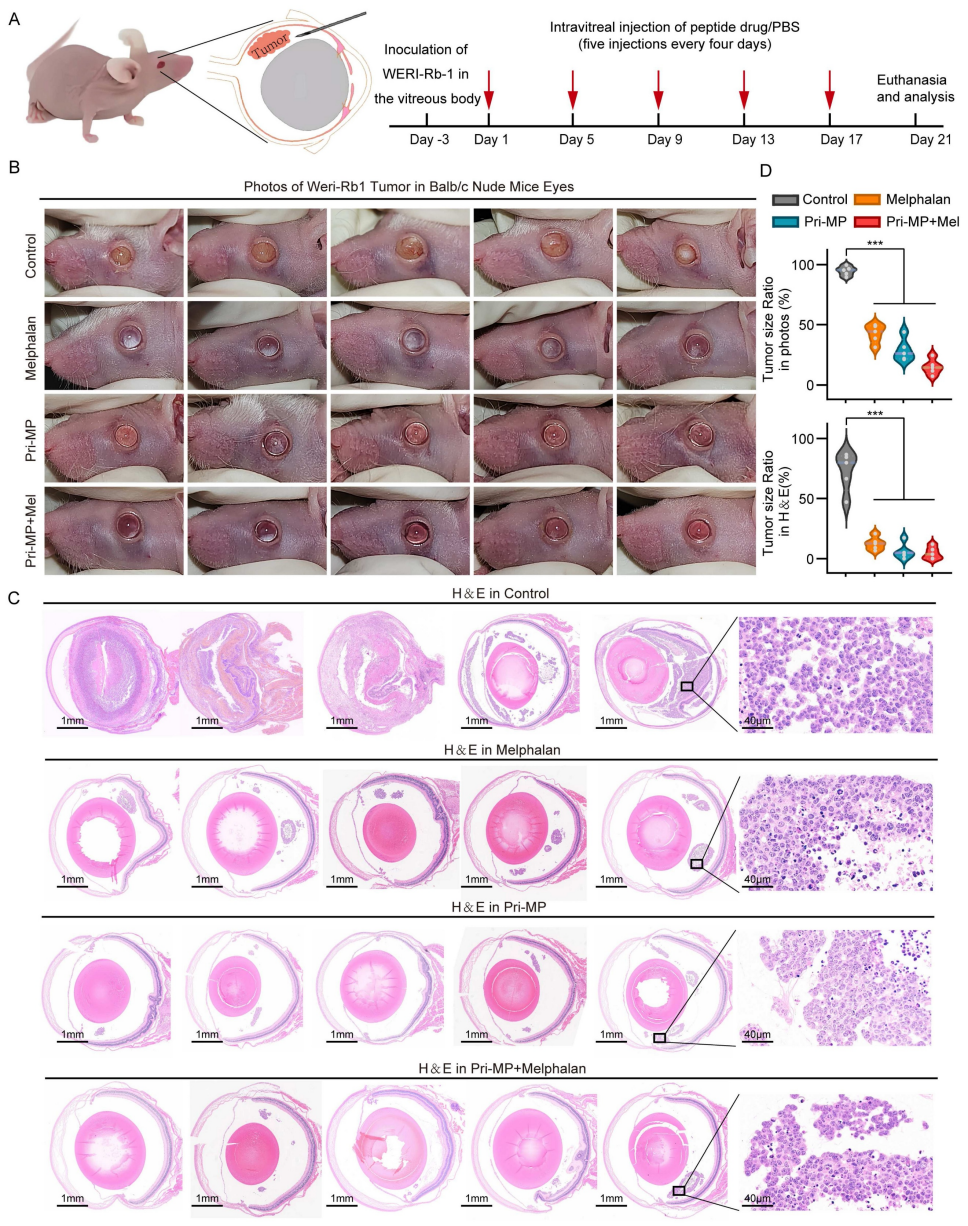
Pri-MP potently suppressed retinoblastoma in a Mouse Orthotopic RB Model. (A) Schematic representation of the treatment protocol. We established a mouse orthotopic RB model by injecting WERI-Rb1 cells into the vitreous cavity of nude mice, which were then randomly divided into four groups (n = 5 per group). Subsequently, the mice were subjected to intravitreal injections of PBS, melphalan (0.5 μg), Pri-MP (0.4 μg), or a combination of Pri-MP and melphalan on days 1, 5, 9, 13, and 17. (B) Images of WERI-Rb1 xenograft models. (C) H&E staining of WERI-Rb1 tumor sections (scale bar: 1mm and 40μm). (D) Quantitative representation of the tumor area as a proportion of the total ocular area in photographs and ocular sections. ***, p < 0.001.

To evaluate the *in vivo* efficacy of Pri-MP, we inoculated cultured WERI-Rb1 cells into the vitreous cavity of nude mice to establish an orthotopic RB model. Mice were then randomly assigned to four groups (n = 5 per group): Control, Melphalan, Pri-MP, and Combination therapy (Pri-MP + Melphalan). Of note, the positive control used in this study, Melphalan, is an alkylating chemotherapeutic agent that inhibits tumor cell proliferation by interfering with DNA synthesis and repair. While it is considered a relatively safe chemotherapy drug that can provide ocular protection, its overall globe salvage rate among patients receiving melphalan-assisted chemotherapy remains at 66.4%^35^. Therefore, investigating the combinatorial antitumor potential of Pri-MP and melphalan holds significant promise. The administration route and dosage were intravitreal injection of melphalan (0.5 µg) and/or Pri-MP (0.4 µg) every four days for a total of five treatment cycles (Fig. 4A). At the experimental endpoint, mice were euthanized, the eyes were photographed *in situ*, and the eyeballs were excised for further analysis.

Photographs of mouse eyes revealed that the Control group presented a large, opaque white area in the eye, indicating severe intravitreal turbidity. In the Melphalan group, some white regions remained visible on the retinal surface. By contrast, no evident tumor growth was observed in either the Pri-MP or the Combination group, especially in the Combination group, where the retina was almost free of white tumor masses. These results suggest that Pri-MP treatment effectively inhibited tumor growth in the orthotopic mouse RB model, outperforming monotherapy with melphalan, and that combined therapy significantly enhances antitumor effects (Fig. 4B).

Next, histological analysis was performed on mouse eyeball sections using H&E staining (Fig. 4C). In the Control group, the tumor region was extensive and had clearly invaded the anterior chamber, extensively disrupting ocular structure. By comparison, melphalan treatment significantly reduced the overall tumor area, confining it to the posterior chamber. Similarly, the tumor area in the Pri-MP and combination groups was markedly reduced; in some mice from the Combination group, intravitreal tumor tissue was almost undetectable. Quantification of tumor area both in gross photographs and in H&E-stained sections revealed that melphalan and Pri-MP monotherapies each reduced tumor burden by 82.6% and 91.7%, respectively, whereas combined therapy led to a 93.6% reduction in tumor area (Fig. 4D). These findings indicate that Pri-MP significantly inhibits tumor growth in a mouse RB orthotopic model.

**Figure 5:**
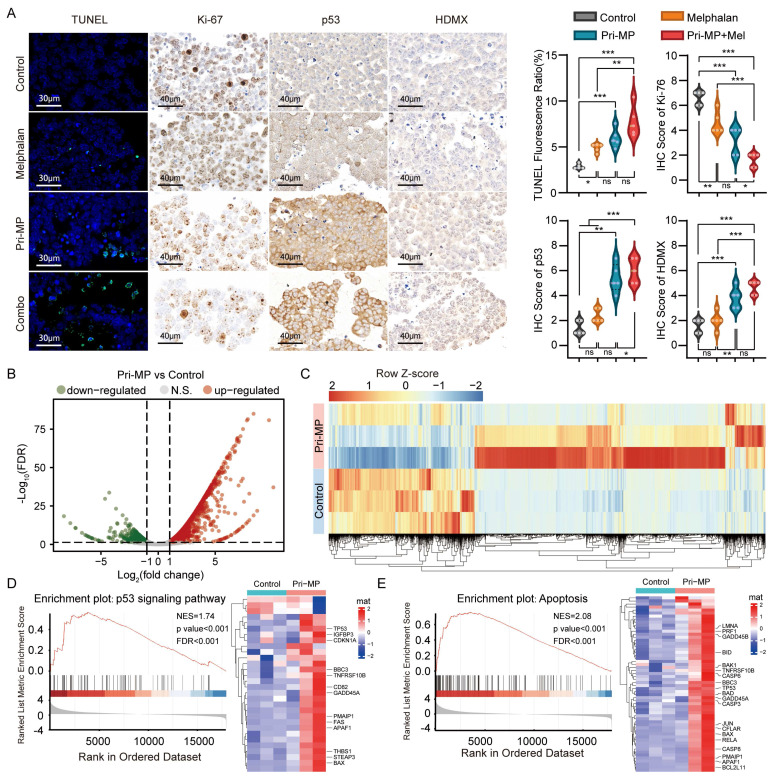
Pri-MP elicits antitumor effects *in vivo* through activation of the p53 signaling pathway. (A) TUNEL assay (scale bar: 30 μm) and quantification of WERI-Rb1 tumor sections. Representative IHC images of Ki-67, HDMX, and p53 and statistical analysis (scale bar: 40 μm). (B, C) Volcano plot (B) and heatmap(C) of differentially expressed genes between Pri-MP and the control group. (D, E) GSEA and hierarchical clustering of genes results showing the p53 signaling (D) and Apoptosis(E) pathway differentially expressed in response to Pri-MP and the Control group (n = 3). * p < 0.05, **, p < 0.01, ***, p < 0.001, ns. not significant.

We subsequently performed TUNEL staining on eyeball sections, followed by fluorescence quantification (Fig. 5A). TUNEL positivity in the Melphalan, Pri-MP, and Combination groups was 1.77-, 2.86-, and 4.56-fold higher, respectively, than in the Control group, demonstrating varying degrees of tumor cell apoptosis in all treatment groups. Notably, the combination therapy induced a significantly greater proportion of apoptotic cells compared to Melphalan alone(p<0.01). In parallel, immunohistochemical (IHC) staining and quantitative analysis of the proliferation marker Ki-67 in tumor tissues (Fig. 5A) revealed that Ki-67 expression levels decreased by about 30.3% and 51.5% in the Melphalan and Pri-MP groups, respectively, relative to the control. Remarkably, the combination group exhibited a 75.8% reduction in Ki-67 expression compared to the control, and a 65.2% decrease relative to the Melphalan group, both reaching statistical significance.

Taken together, although the combination therapy exhibited a trend toward enhanced tumor suppression compared to melphalan monotherapy, the reduction in overall tumor burden did not reach statistical significance, potentially due to the limited duration of treatment. Nonetheless, given the marked increase in tumor cell apoptosis and the significant suppression of proliferative activity observed in the combination group, we conclude that the co-administration of Pri-MP and melphalan possesses greater therapeutic potential for tumor inhibition. Future studies involving varied dosing regimens and prolonged treatment durations are warranted to evaluate the pharmacodynamic interactions systematically, optimize administration strategies, and assess the overall therapeutic efficacy of this combination, thereby providing a theoretical and experimental foundation for the clinical translation of intraocular chemotherapy 36.

To further investigate the mechanism underlying Pri-MP's antitumor effects, we performed IHC staining to detect p53 and its negative regulator HDMX. As shown in Fig. 5A, p53 expression in the Melphalan, Pri-MP, and Combination groups increased by 71%, 286%, and 327%, respectively, relative to the Control group, consistent with our *in vitro* studies. Moreover, we conducted an RNA-seq analysis of RB orthotopic tumor tissues in mice under treatment. Differential expression analysis revealed that compared with the control group, 2,684 genes in the Pri-MP group were significantly altered (p < 0.05, |Log2FC| > 1), including 1,787 genes that were upregulated and 897 that were downregulated (Fig. 5B&C). GSEA showed that genes related to the p53 signaling pathway (Fig. 5D) and apoptosis (Fig. 5E) were significantly enriched following Pri-MP intervention. These results indicate that Pri-MP potently antagonizes HDMX, thereby activating the p53 pathway and inducing apoptosis in RB tumor cells, ultimately exerting a robust antitumor effect in the mouse orthotopic RB model.

### Systemic safety and pharmacokinetics of Pri-MP

**Figure 6:**
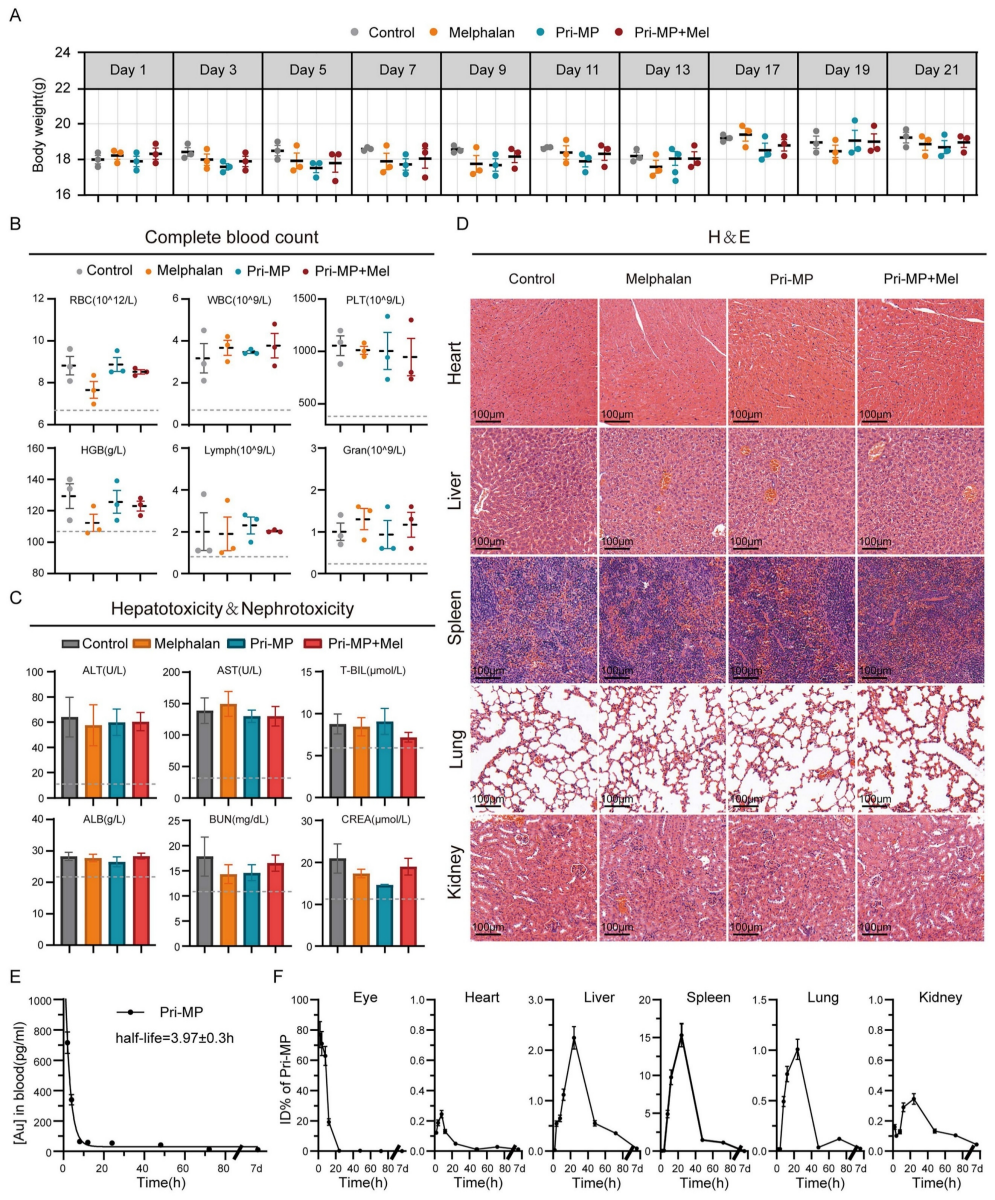
Biosafety of Pri-MP in BALB/c mice. (A) Analysis of body weight variations among four distinct groups of mice. (B) Evaluation of hematological parameters, including red blood cells (RBC), white blood cells (WBC), platelets (PLT), hemoglobin (HGB), lymphocytes (Lymph), and granulocytes (Gran), in murine blood following specified treatments (n = 3). (C) Assessment of Pri-MP-induced hepatotoxicity through measurements of aspartate transaminase (AST), alanine aminotransferase (ALT), total bilirubin (T-BIL), and albumin (ALB). Nephrotoxicity was evaluated by determining blood urea nitrogen (BUN) and creatinine (CREA) levels (n = 3). (D) Examine Pri-MP-induced potential cardiotoxicity, hepatotoxicity, splenic toxicity, pulmonary toxicity, and nephrotoxicity via hematoxylin and eosin (H&E) staining (scale bar: 100 μm). (E) Pharmacokinetic profile of Pri-MP pharmacokinetics using ICP-MS. (F) Biodistribution of Pri-MP in mice at different time points post-treatment (including eyes, heart, liver, spleen, lung, and kidney) using ICP-MS.

Assessment of *in vivo* biosafety is a critical step in drug development and clinical translation, making it essential to explore and evaluate the systemic safety of Pri-MP. First, we closely monitored the body weight of mice throughout the treatment period. Overall, body weights in all four groups gradually increased over time, with no significant difference between the Melphalan group, the Pri-MP group, and the Control group. Even in the Combination group, mice did not experience significant weight loss, indicating that Pri-MP does not adversely affect normal weight gain (Fig. 6A).

At the end of treatment, complete blood counts and serum biochemistry tests were performed to evaluate hematologic toxicity and hepatic/renal function. Hematological data showed that neither Pri-MP alone nor in combination with melphalan caused any significant changes in red blood cells, hemoglobin, white blood cells, or platelets (Fig. 6B), indicating that neither treatment regimen induced hematologic toxicity. Liver function tests revealed that alanine aminotransferase and aspartate aminotransferase levels in mice treated with melphalan alone, Pri-MP alone, or both agents together did not significantly deviate from the Control group, and albumin and bilirubin levels remained within normal ranges. Similarly, renal function tests showed that neither Pri-MP monotherapy nor combination therapy caused any significant alterations in renal function (Fig. 6C). Furthermore, H&E staining of major organs demonstrated no drug-related toxicities in the lungs, liver, kidneys, or other organs in either the Pri-MP or the Combination groups (Fig. 6D).

To evaluate whether intravitreal administration of Pri-MP induces retinal toxicity, we performed electroretinography (ERG) assessments following injection of Pri-MP alone or in combination with melphalan at both therapeutic and supratherapeutic doses. Retinal function was assessed by measuring a-wave and b-wave amplitudes under dark-adapted (scotopic) and light-adapted (photopic) conditions (Fig. S14A), reflecting rod and cone activity, respectively 37. Across all tested doses, Pri-MP did not elicit any significant reductions in either a-wave or b-wave amplitudes (Fig. S14B). These findings indicate that Pri-MP has minimal impact on retinal neuronal function and demonstrate its favorable intraocular safety profile, supporting its potential for clinical translation.

To further confirm that Pri-MP undergoes clearance *in vivo*, we used ICP-MS to measure the dynamic metabolic processes and organ distribution of Pri-MP following intravitreal injection in healthy BALB/c mice. Time-course ICP-MS analysis of blood pharmacokinetics revealed that the drug was nearly eliminated from the bloodstream by 24 hours (t1/2 = 3.97 ± 0.3 h; Fig. 6E). According to the time-dependent percentage-injected-dose data of ^197^Au in various organs, over 50% of ^197^Au was cleared from the eye by 12 hours post-injection, and virtually all was cleared by 24 hours (Fig. 6F). Notably, no significant ^197^Au residuals were detected in the heart, liver, spleen, kidney, or lung one week after injection (Fig. 6F). These results indicate that Pri-MP is effectively metabolized and cleared *in vivo*. In summary, Pri-MP exhibits a favorable safety profile and excellent clearance properties when administered *via* intravitreal injection, either alone or in combination with melphalan.

## Discussion

In this study, we systematically investigated a prion-like self-assembling peptide prodrug (Pri-MP) and demonstrated its potent antitumor efficacy against retinoblastoma (RB). First, single-cell RNA sequencing and bioinformatic analyses revealed that RB cells exhibit robust macropinocytic activity *via* the Rac1-PAK1 signaling axis, providing a distinctive therapeutic window for tumor-specific drug delivery. Guided by this molecular insight, we designed an HDMX-binding peptide (MP) that competitively occupies the p53-binding interface on HDMX. By employing Au(I)-mediated reversible assembly, MP was incorporated into a prion-like microprotein (Pri-MP), which preferentially entered RB cells through macropinocytosis, thereby achieving selective intracellular accumulation. *In vitro* assays showed that Pri-MP reactivated p53 signaling, induced cell-cycle arrest, and triggered apoptosis in RB cells harboring wild-type TP53 but had negligible effects on normal retinal cells. In an orthotopic RB mouse model, intravitreal injection of Pri-MP significantly suppressed tumor burden and cooperated with standard-of-care melphalan to further boost antitumor effects, all while maintaining a favorable safety profile. These findings firmly establish Pri-MP as a promotable RB-targeting agent that not only overcomes the toxicity barriers seen in previous attempts at p53 pathway modulation but also offers clear advantages in selective tumor cell uptake.

It has been shown that retinoblastoma (RB) cell proliferation and tumor formation are primarily dependent on high-level expression of MDM2, rather than MDM4(HDM4/HDMX), and that this dependency is largely mediated through the maintenance of elevated MYCN expression rather than inhibition of p53-mediated apoptosis 12. However, our study reveals that targeted inhibition of HDMX is also sufficient to activate p53 signaling and elicit potent anti-tumor responses. These findings complement and expand the current understanding of the HDM2/HDMX-p53 regulatory axis in retinoblastoma. Notably, our findings parallel those of Aubry *et al.* 37, who demonstrated that the neddylation inhibitor MLN4924 synergizes with melphalan or topotecan by activating a p53/MYCN-driven pro-apoptotic network, leading to complete tumor regression without ocular toxicity. Similarly, Pri-MP, particularly in combination with melphalan, upregulates the p53 pathway and enhances apoptosis in our model. Although the reduction in tumor volume did not reach statistical significance, the shared engagement of the p53 axis highlights a convergent therapeutic mechanism. These observations support the potential of combination strategies that reinforce intrinsic tumor suppressor pathways while maintaining safety. Our work holds substantial promise for advancing RB treatment, an area where no widely accepted, clinically effective targeted therapies are currently available. The success of targeted therapy in other malignancies demonstrates that small-molecule drugs or biological agents directed against critical oncogenic pathways can markedly reduce systemic toxicity while improving therapeutic efficacy 38, 39. This concept is highly relevant to RB, where standard chemotherapeutic regimens risk significant ocular side effects and systemic toxicity. The p53-HDMX axis represents a particularly vital target in RB, as most RB tumors still retain functional p53 but suffer from the inhibitory effects of overexpressed HDMX 8. Here, Pri-MP directly antagonizes HDMX, liberating p53 to reestablish its tumor-suppressive functions. Since p53 lies at the core of DNA damage response and apoptosis, this pathway is integral to preventing tumor progression in RB, thereby suggesting that an HDMX-targeting therapeutic, such as Pri-MP, could transform the RB treatment paradigm. By effectively restoring p53 function in a highly specific and safe manner, our study underscores the pivotal role of p53-HDMX blockade and its translational potential as a future cornerstone in RB therapy.

Another central contribution of this work lies in its use of a prion-like nanostructure to achieve tumor-selective drug delivery. The effective accumulation of therapeutic agents in tumor cells, coupled with minimal uptake by healthy tissues, has long been recognized as a key strategy for maximizing antitumor activity while sparing normal physiology 40. Here, Pri-MP exploits the pronounced macropinocytosis in RB cells, an approach that has shown remarkable success in several other tumors exhibiting high macropinocytic flux (e.g., pancreatic ductal adenocarcinoma) 41. By adapting prion protein-inspired neurotropism and endocytic mechanisms, Pri-MP enhances RB cell targeting, thereby improving therapeutic indices and minimizing off-target toxicities. This strategy aligns with emerging data in other tumor types, where tumor-specific cellular uptake pathways are leveraged to develop “smart” drug carriers that significantly improve efficacy/safety ratios 42. Consequently, our findings reinforce that rationally designed nano-delivery systems targeting RB cells can be instrumental in achieving more effective and less toxic therapies.

In summary, this work provides a solid foundation for a novel p53-reactivation strategy in retinoblastoma, offering a clear rationale for combining Pri-MP with existing chemotherapies to improve eye-preserving treatments and potential patient outcomes. Despite these promising results, certain limitations remain. Our approach still relies on intravitreal injection for drug delivery, which, although clinically feasible, is invasive and may be optimized in the future. A noninvasive eye-drop formulation or other sustained-release systems could further enhance patient compliance and safety while maintaining therapeutic efficacy. Nonetheless, the development of Pri-MP not only represents a promising step forward in RB care but also exemplifies a broader strategy applicable to other solid or neuroectodermal tumors. By integrating precise molecular targeting with sophisticated nanoscale delivery, we contribute to the growing body of evidence that prion-like or biomimetic nanotechnology can serve as a powerful platform for anticancer drug development. We anticipate that continued innovation along these lines will significantly advance RB therapy and reshape the landscape of targeted cancer treatments.

## Supplementary Material

Supplementary figures and methods.
