# Supplementary material for: Bringing p53 Back: A Prion-Powered Attack on Retinoblastoma
Source: Int J Biol Sci. 2025 Sep 22;21(13):5975–88. doi: 10.7150/ijbs.113116 (PMC12510174; doi:10.7150/ijbs.113116)
Supplement: Supplementary file 1 — Supplementary figures and methods. [file ijbsv21p5975s1.pdf]

Supplementary materials

Bringing p53 Back: A Prion-Powered Attack on Retinoblastoma

Yuyan Ma[1,†], Siqi Yan[2,3,4,†], Weiming You[2,4], Peili Wang[1], Wangxiao He[1,*], Yu Yao[1,*], Xiaoqiang Zheng[2,3,*]

1. Department of Medical Oncology, The First Affiliated Hospital of Xi'an Jiaotong University, Xi'an 710061, China.

2. Department of Hepatology, The Second Affiliated Hospital of Xi'an Jiaotong University, Xi'an 710004, China.

3. Institute for Stem Cell & Regenerative Medicine, The Second Affiliated Hospital of Xi'an Jiaotong University, Xi'an 710004, China.

4. National & Local Joint Engineering Research Center of Biodiagnosis and Biotherapy, The Second Affiliated Hospital of Xi'an Jiaotong University, Xi'an 710004, China.

[†] These authors contributed equally.

[*] Corresponding authors:

Email: hewangxiao5366@xjtu.edu.cn (W. He)

Email: yaoyu123@xjtufh.edu.cn (Y. Yao)

Email: zhengxiaoqiang@xjtu.edu.cn (X. Zheng)

1. Supplementary Figures

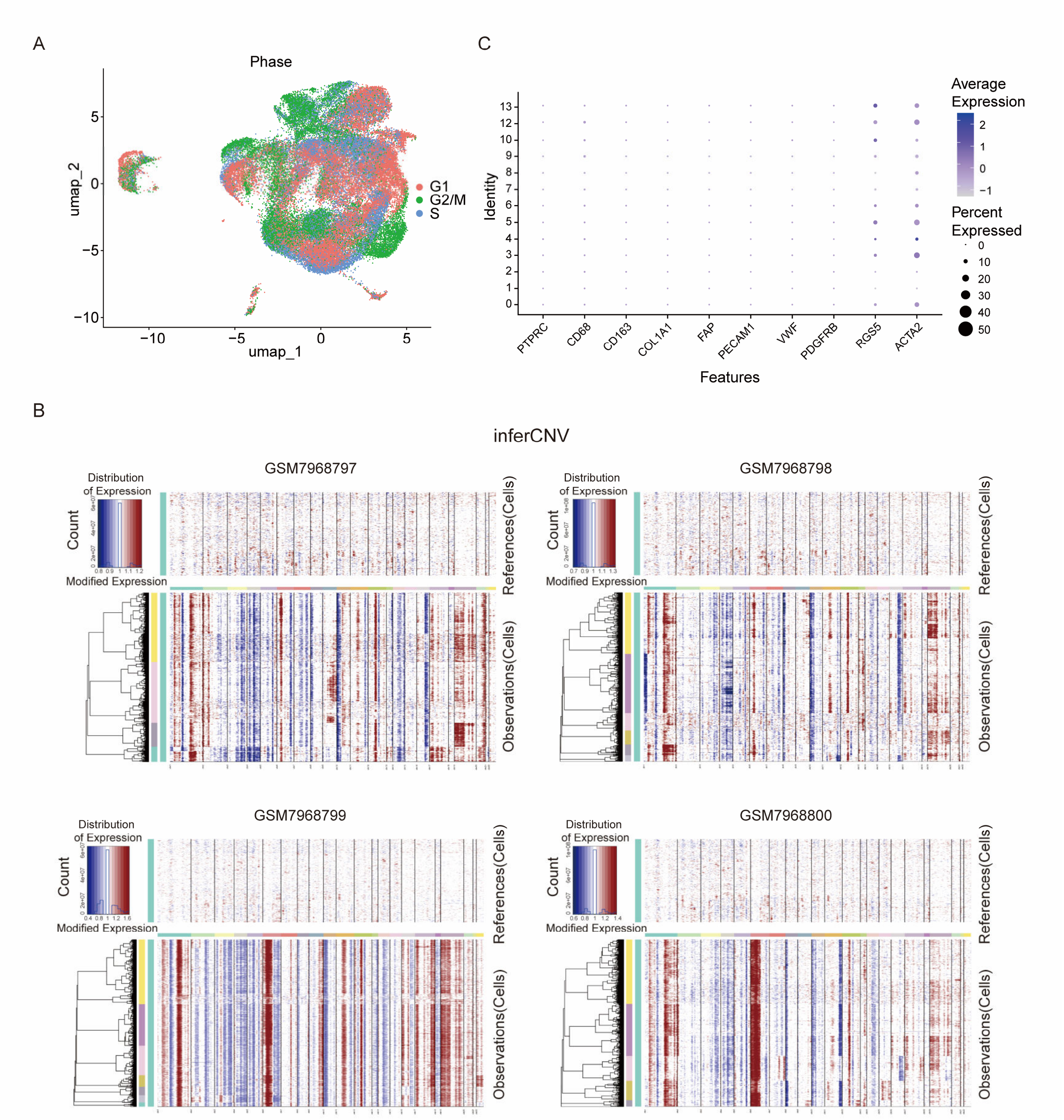

Figure S1. Integrated single-cell transcriptomic analysis of retinoblastoma (RB) samples. (A) Distribution of cell cycle phases across clusters after batch-effect correction using Harmony. (B) Identification of malignant cells from four RB samples (GSM7968797, GSM7968798, GSM7968799, GSM7968800, from GSE249995) based on copy number variation (CNV) inference by inferCNV, with non-neoplastic retinal cells (GSE196235) serving as the reference. (C) The expression profiles of canonical immune markers (PTPRC/CD45, CD68, CD163) and stromal markers (COL1A1, FAP, PECAM1/CD31, VWF, PDGFRB, RGS5, ACTA2) were analyzed within annotated "retinoblastoma" clusters.

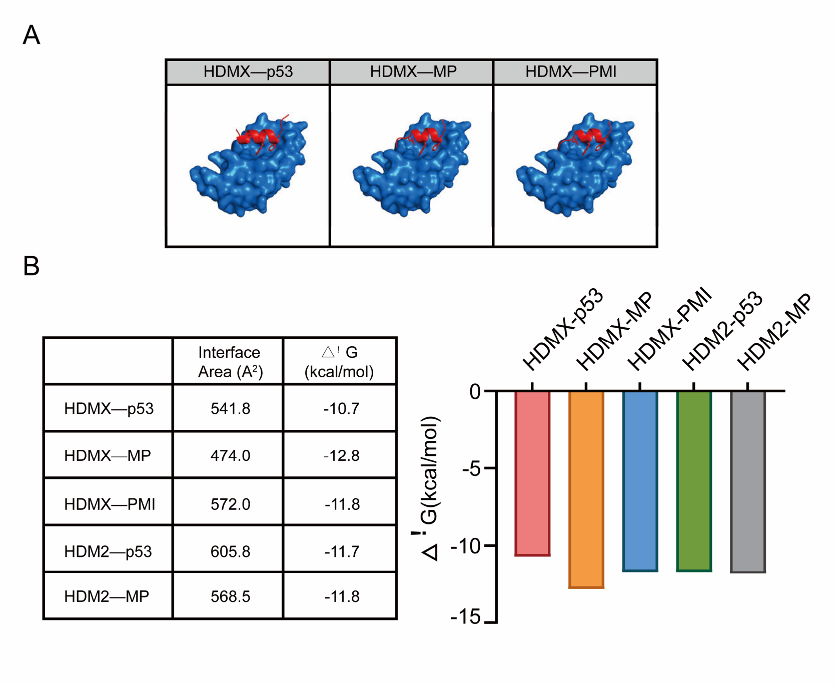

	Interface Area (A²)	△G (kcal/mol)
HDMX—p53	541.8	-10.7
HDMX—MP	474.0	-12.8
HDMX—PMI	572.0	-11.8
HDM2—p53	605.8	-11.7
HDM2—MP	568.5	-11.8

Figure S2. Evaluation of the binding ability of Pri-MP and HDMX. (A)Structural modeling of peptide-HDMX complexes via AlphaFold-Multimer deep learning framework. (B) Comparative binding free energy (ΔG, kcal/mol) analysis of HDMX/HDM2 complexes with p53 and engineered peptides.

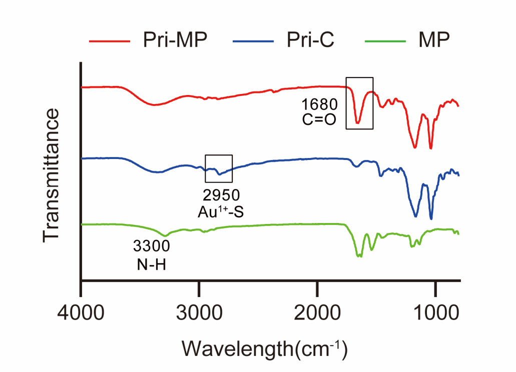

Figure S3. Infrared spectroscopy graph of MP and Pri-MP (Wavelength Scanning Range: 800–4000 nm).

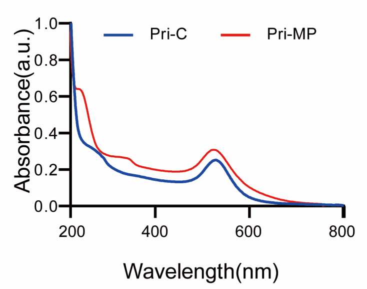

Figure S4. The UV-Vis absorption spectrum of Pri-C and Pri-MP.

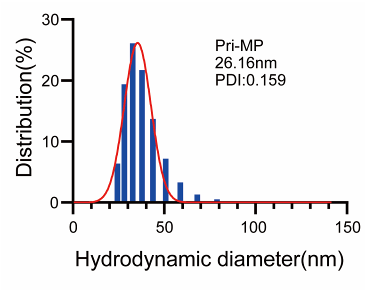

Figure S5. Hydrodynamic diameters of Pri-MP, as measured by dynamic light scattering.

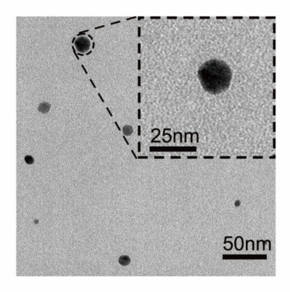

Figure S6. TEM images of Pri-MP.

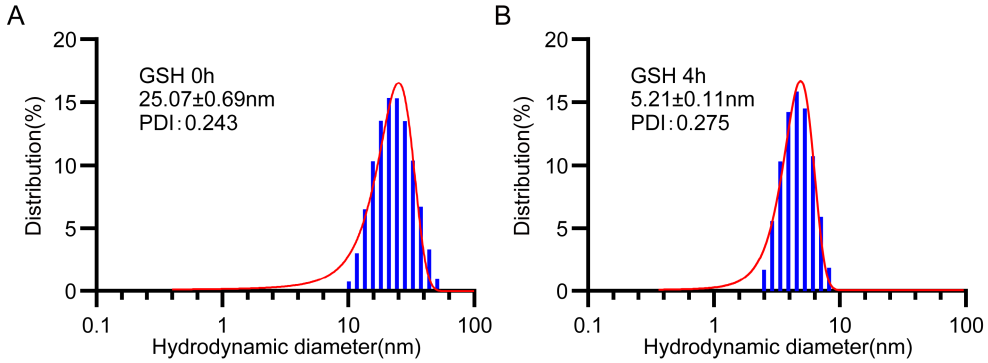

Figure S7. Hydrodynamic distributions of Pri-MP before (A) and after (B) 10 mM GSH incubation, which were measured by dynamic light scattering.

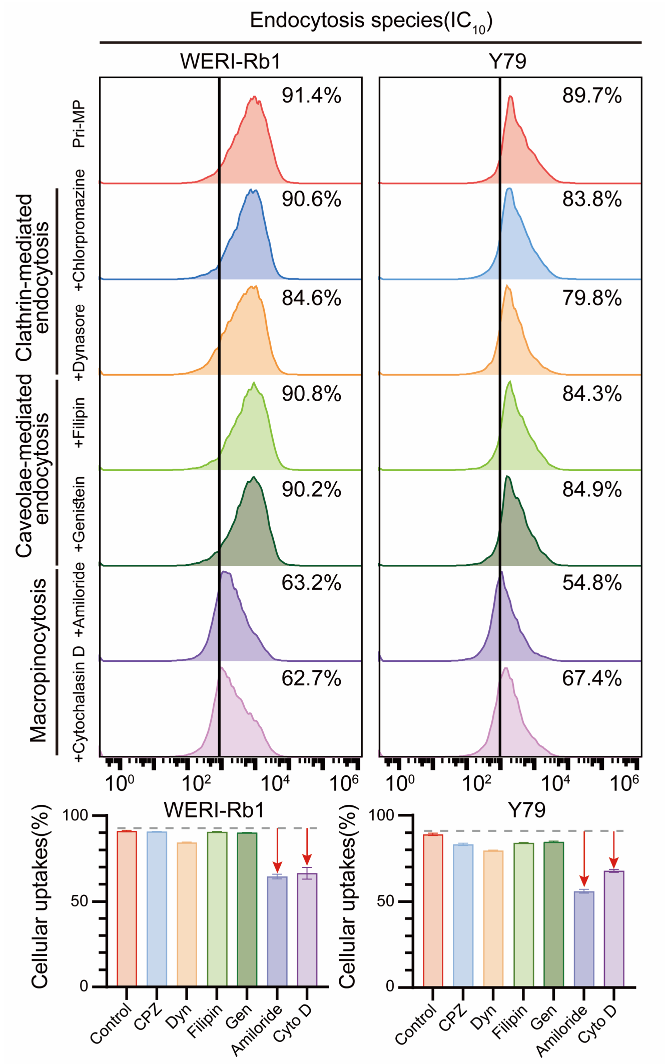

Figure S8. Flow cytometry analysis of Pri-MP cellular internalization mechanisms in different concentrations. IC_{10} represents the concentration of chlorpromazine or other inhibitors that results in a 10% inhibition of cell growth compared to untreated controls.

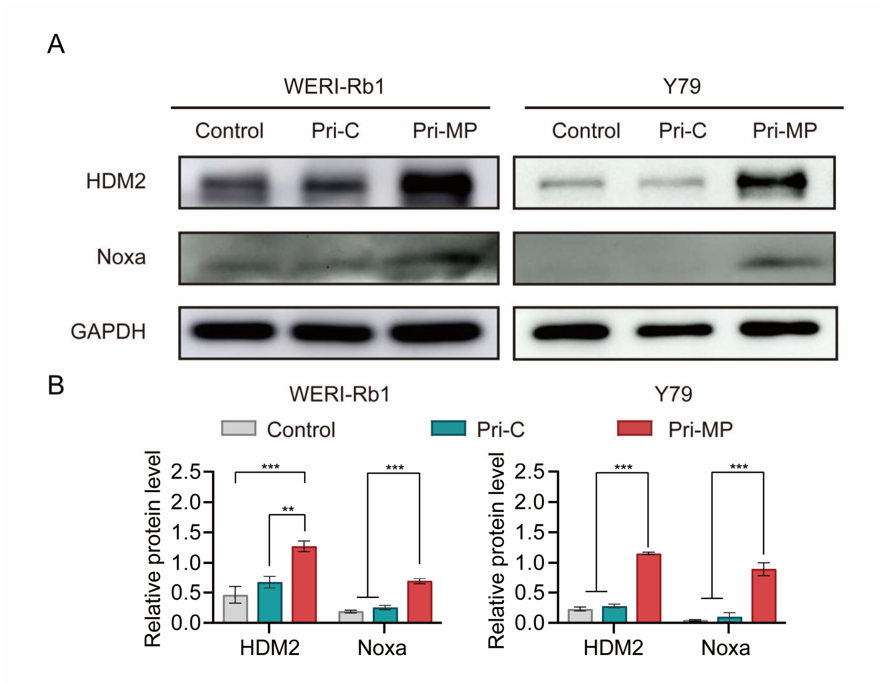

Figure S9. Immunoblot analysis (A) and quantitative assessment (B) of HDM2 and Noxa protein expression in WERI-Rb1 and Y79 cells (n=3). **, $p < 0.01$, ***, $p < 0.001$.

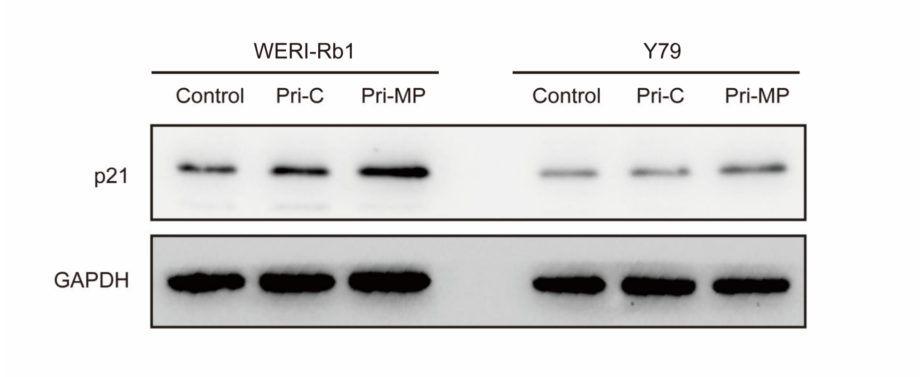

Figure S10. Western blot analysis of p21 expression in WERI-Rb1 and Y79 cells under identical exposure conditions.

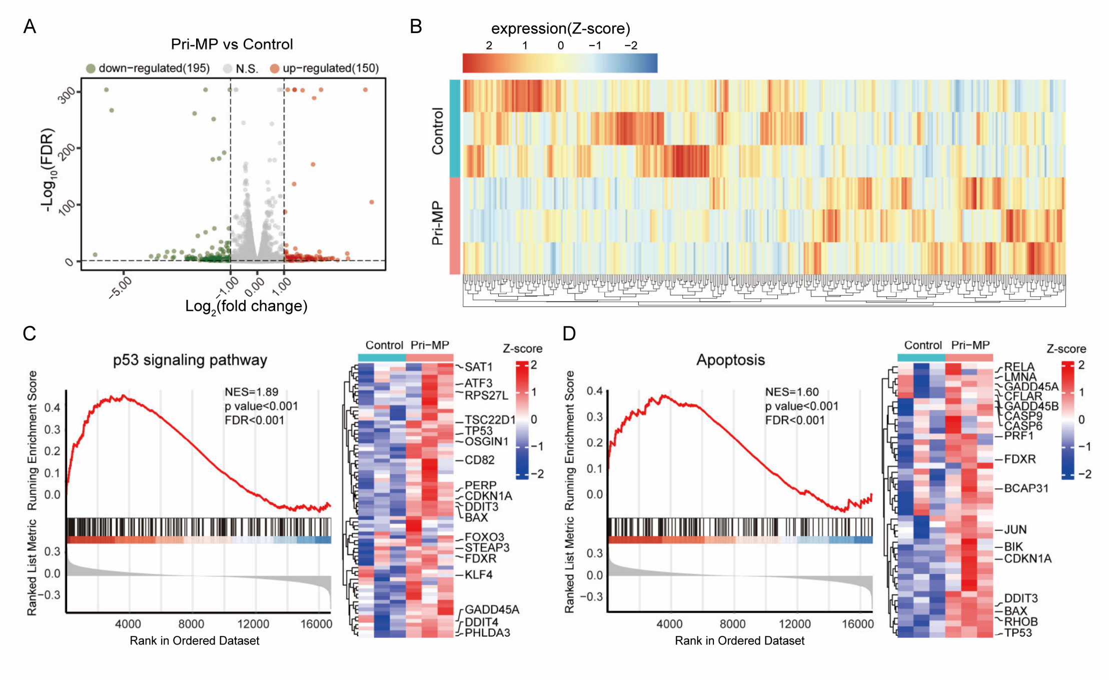

Figure S11. RNA-seq analysis of tumor cells following treatment. (A, B) Volcano plot (A) and hierarchical clustering (B) of differentially expressed genes between Pri-MP and the control group. (C, D) GSEA and hierarchical clustering of genes results showing the p53 signaling(C) and apoptosis pathway(D) differentially expressed in response to Pri-MP and the control group in vitro. (n = 3).

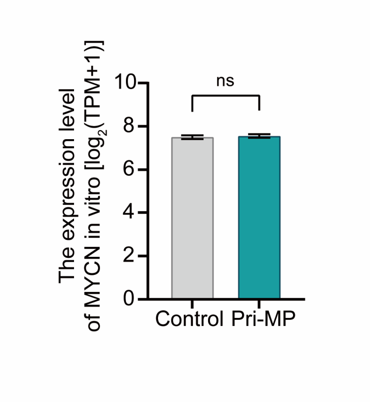

Figure S12. The expression level of MYCN in RNA-seq in vitro (n = 3).

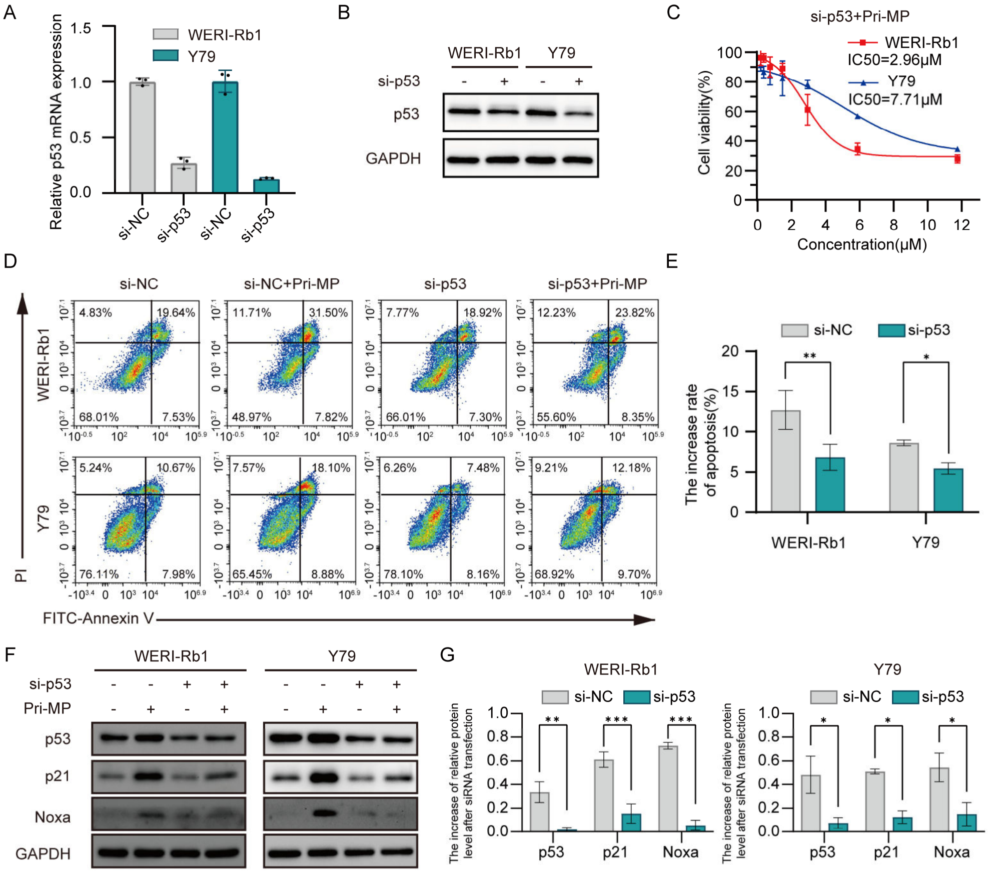

Figure S13. Pri-MP's tumor-suppressive effect is critically dependent on p53 activation. (A, B) The qPCR (A) and Western blot (B) results demonstrated that p53 mRNA and protein expression were suppressed in both WERI-Rb1 and Y79 cell lines. (C) Dose-dependent growth inhibition of WERI-Rb1 and Y79 cells transfected with si-p53. (D, E) Apoptosis in si-p53–transfected WERI-Rb1 and Y79 cells following Pri-MP treatment was assessed by flow cytometry (D), and the proportion of apoptotic cells was quantified and presented as bar graphs (E). (F, G) WERI-Rb1 and Y79 cells were transfected with si-p53 or si-NC, followed by Pri-MP treatment. (F) Western blot analysis of p53 and its downstream effectors p21 and NOXA. (G) Comparative analysis of protein upregulation across transfection groups following Pri-MP treatment (n = 3). $P < 0.05$; $^*P < 0.01$; $^{**}P < 0.001$.

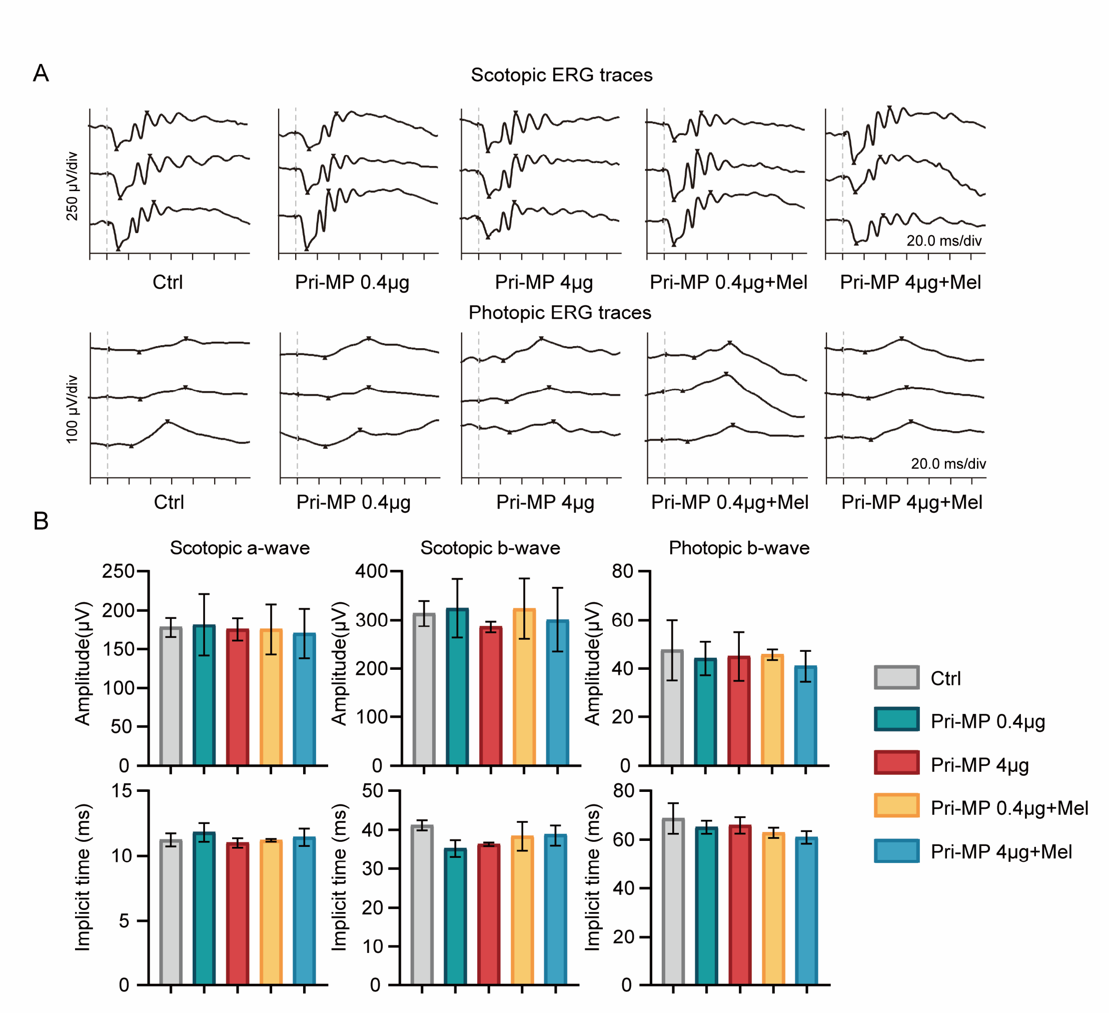

Figure S14. Electroretinographic assessment of retinal function following intravitreal administration of Pri-MP. (A) Representative scotopic and photopic ERG waveforms were recorded in mice treated with various doses of intravitreal Pri-MP or combination therapy. (B) Quantitative analysis of a-wave and b-wave amplitudes revealed no significant differences among treatment groups (n=3).

2. Materials and Methods

Single-cell RNA sequencing data acquisition and preprocessing

The single-cell RNA sequencing (scRNA-seq) dataset GSE249995 was obtained from the Gene Expression Omnibus (GEO) database, comprising intraocular tissue samples from 4 retinoblastoma (RB) patients. Principal component analysis (PCA) was performed on variable genes. Cells were clustered using the Louvain algorithm and visualized *via* Uniform Manifold Approximation and Projection (UMAP). This process partitioned the cells into 16 distinct clusters. Cross-sample integration confirmed high consistency in cluster distribution across patients. Cell clusters were annotated using canonical marker genes derived from published retinal single-cell atlases. The Wilcoxon rank-sum test was employed to analyze differential gene expression between RB-like cells and normal intraocular cells.

Peptide design and peptide-protein docking

The amino acid sequence of protein HDMX was meticulously retrieved from the Protein Data Bank (PDB). The design of the MP Peptide was implemented with utmost care using ProteinMPNN. Subsequent prediction of binding conformations between the protein and the designed peptide was performed through the AlphaFold Server. Interaction parameters, including contact surface area and binding energy, were calculated with precision via the PDBePISA web interface. Structural visualization and analysis of all protein-ligand complexes were conducted with meticulous attention to detail using the PyMOL molecular graphics system.

Synthesis of peptide MP

The MP peptide was precisely synthesized on MBHA resin using a CS Bio 336X automated peptide synthesizer, following Fmoc solid-phase synthesis protocols. The purification of MP to homogeneity was achieved through preparative C18 reversed-phase high-performance liquid chromatography (HPLC), using acetonitrile and water with 1/1000 TFA as the reagent. This process, verified by electrospray ionization mass spectrometry (ESI-MS) and HPLC, ensured high peptide quality.

Self-assembly of Pri-MP

First, 2 mg of MP was completely dissolved in a solution containing 500 μL ethanol and 1.25 mL ddH_2O. After that, an aqueous solution of tetrachloroauric acid (HAuCl4·XH_2O, 1 mL, 10 mM) was mixed with 500 μL NH2-PEG-SH (MW: 2000, 4 mg/mL in deionized water) and 2.25 mL HEPES (100 mM, pH 7.0). It was mixed with a solution containing 2.25 mL of deionized water and 2.25 mL of HEPES (100 mM, pH 7.0) and sonicated for 10 minutes. Finally, excess reactants were removed through a dialysis tube and washed twice with distilled water.

Characterization of Pri-MP

The dried samples were examined with a 120 kV transmission electron microscope (TEM) to determine particle size. The hydrodynamic size distribution was determined using dynamic light scattering (DLS) measurements with a Malvern Zetasizer Nano ZS system. Samples underwent 15 minutes of sonication before analysis. Subsequently, the samples were positioned in the measuring cell and given a 2-minute equilibration period. The hydrodynamic diameter and number distribution of the samples were determined from three independent runs. Fourier transform infrared spectroscopy was employed to characterize the surface chemical structures of Pri-MP and Pri-C. The UV–vis absorption spectra of Pri-MP and MP were measured from 200 to 800 nm using a Shimadzu 3000 spectrophotometer. To test GSH-responsive drug release, Pri-MP was dissolved in PBS buffer (pH 7.4) containing 10 mM glutathione (GSH), and the Pri-MP solution was then centrifuged at 14,000 g, and the hydrodynamic size distribution was determined using DLS measurements.

Cell culture and reagents

Human retinoblastoma cell lines (WERI-Rb1, Y79) and regular ocular cell lines (661W, ARPE-19, HLE-B3, MIO-M1) were obtained from ATCC, while RAW264.7 was acquired from the Cell Bank of the Chinese Academy of Sciences in Shanghai, China. WERI-Rb1, Y79, and MIO-M1 cells were cultured in RPMI-1640 with 10% FBS (Gibco) at 37°C and 5% CO_2. 661W, and RAW264.7 cells used DMEM, HLE-B3 cells used MEM, and ARPE-19 cells used DMEM/F12. Pri-MP was synthesized as previously described. Melphalan was dissolved in DMSO (final concentration <0.1%).

The assessment of cell viability in vitro assays

Cell lines WERI-Rb1, Y79, and 661W were cultured at a density of 1×10^{5} cells/mL in 96-well plates. Following exposure to varying concentrations of Pri-MP or Pri-C, Alamar Blue reagent was introduced to each well and incubated for 4 hours at 37°C. Fluorescence intensity was assessed with a microplate reader at excitation and emission wavelengths of 560 nm and 590 nm, respectively.

The cellular internalization of Pri-MP

To investigate the cellular internalization mechanisms of Pri-MP in WERI-Rb1 and Y79 cells, we employed six inhibitors targeting distinct pathways involved in cellular internalization: chlorpromazine and dynasore for inhibiting clathrin-mediated endocytosis, filipin and genistein for inhibiting caveolae-mediated endocytosis, and amiloride and cytochalasin D for inhibiting macropinocytosis. After treating the cells with the inhibitors for 1 hour, FITC-labeled MP or Pri-MP was introduced to the medium and incubated at 37 °C for 6 hours. After washing the cells with PBS to remove excess drugs, we performed flow cytometry (FCM) analysis.

Cell transfection with p53-targeting siRNA

WERI-Rb1 and Y79 cells were seeded in 24-well plates at a density of 1×10^{5} cells/well. Transient transfection was performed using Lipofectamine™ 3000 reagent. 50 nM human TP53 siRNA or scrambled control siRNA was diluted in Opti-MEM Reduced Serum Medium. Diluted siRNA was combined with Lipofectamine™ 3000 reagent at a 1:1 ratio (v/v) and incubated for 15 min at room temperature. The siRNA-lipid complexes were added dropwise to cells in antibiotic-free medium. Cells were incubated at 37°C in 5% CO_2 for 24 h prior to subsequent analyses. The sequences of siRNA were used: si-p53 sense: GGAAGACUCCAGUGGUAAU, si-p53 antisense: AUUACCACUGGAGUCUUCC.

RT-qPCR Assay:

Total RNA was extracted using StarSpin Fast Cell RNA Kit (GenStar, P130-01). Gene expression changes relative to GAPDH were calculated using the ΔΔCT method. The foll owing primer sequences were used: GAPDH Forward: 5'-GGAGCGAGATCCCTCCAAAAT-

3’; GAPDH Reverse: 5'-GGCTGTTGTCATACTTCTCATGG-3'; TP53 Forward: 5'-TTCCTG AAAACAACGTTCTGTC-3'; TP53 Reverse: 5'-AACCATTGTTCAATATCGTCCG-3.

Apoptosis and cell cycle analysis

Apoptosis was evaluated by flow cytometry using the Annexin V-FITC/PI Apoptosis Kit (BD Biosciences). Cells subjected to different treatments were collected, washed, and resuspended at a final concentration of 1×10^6 cells/mL. One hundred microliters of the solution (1×10^5 cells) were transferred to a 5-mL culture tube, adding 5 μL of FITC Annexin V and 5 μL of PI. After gentle vortexing and a 15-minute incubation in the dark at room temperature, 400 μL of 1× binding buffer was added to the tube, and the cells were analyzed using an FCM.

For cell cycle analysis, cells were first serum-starved for 12 hours and then treated with Pri-MP or Pri-C. Next, they were harvested, washed twice in PBS, and fixed in 70% ethanol on ice for at least 30 minutes. After that, they were stained with a PI solution (50 μg/mL PI, 50 μg/mL RNase A). A flow cytometer analyzed cell cycle distributions based on DNA content.

Western blotting

The retinoblastoma cell lines WERI-Rb1/Y79 were initially grown in a 6-well plate and subjected to various drug treatments. Post-treatment, cell lysates were prepared using RIPA buffer with protease inhibitors. Total protein was quantified using a BCA assay and subsequently denatured for downstream applications. These proteins were then separated on an SDS-PAGE gel and transferred to a PVDF membrane using a wet transfer system. Following this, the membrane was blocked with 5% BSA and then incubated with primary and secondary antibodies. Primary antibodies against HDMX (Proteintech, 17914-1-AP; 1:1000), HDM2 (Abcam, ab259265; 1:1000), p53 (Santa Cruz Biotechnology, sc-126; 1:1000), p21(Proteintech, 10355-1-AP; 1:1000), PUMA (Proteintech, 55120-1-AP; 1:500), and Noxa (Abcam, ab13654; 1:500) were incubated overnight at 4°C. GAPDH (Proteintech, 10494-1-AP; 1:5000) served as a loading control. Finally, the results were quantified using ImageJ software.

Mouse Orthotopic RB Model

The study utilized mice obtained from the Laboratory Animal Center at Xi'an Jiaotong University. All animal experiments complied with institutional guidelines and were approved by the Laboratory Animal Center of Xi'an Jiaotong University (approval number 2021-416).

Four-week-old BALB/c nude mice received intravitreal injection of 5×10^5 WERI-Rb1 cells in 3 μL PBS. Following the establishment of the model, the mice were randomly assigned to four distinct groups. Therapeutic agents were administered *via* intravitreal injection using a 33-gauge needle, following a treatment schedule of one dose every four days for a total of five cycles.

H&E and immunohistochemistry

The eyes were excised, fixed in 4% paraformaldehyde, and paraffin-embedded. Then, 4-μm-thick sections were prepared for immunohistochemistry staining. Following dewaxing and hydration, tissue sections were treated with Triton X-100 for 20 minutes to permeabilize them, followed by a PBS wash. The tissue sections were blocked with goat serum for 2 hours. After blocking, tissue sections were incubated with the primary antibody overnight at 4°C, followed by incubation with an HRP-conjugated secondary antibody for 1.5 hours. Image acquisition was performed using an automated slide scanner, and quantitative histomorphometric analysis was conducted using ImageJ software. The antibodies used for immunohistochemistry: Ki-67(CST, D358,1;400), p53 (Proteintech, 10442-1-AP; 1:100), and HDMX (Proteintech, 17914; 1:250).

Terminal deoxynucleotidyl transferase dUTP Nick End Labeling (TUNEL) Staining.

Apoptosis was detected using the TUNEL assay according to the manufacturer's protocol (Roche, No. 11684795910). Tissue sections were deparaffinized, rehydrated, and treated with proteinase K (20 μg/mL) for 15 min at 37°C. After washing with PBS, sections were incubated with a TUNEL reaction mixture (enzyme solution: label solution = 1:9) for 1 hour at 37°C in a humidified chamber. Nuclei were counterstained with DAPI (1 μg/mL).

Transcriptome Analysis

Collected cells and excised tumor tissues were snap-frozen in liquid nitrogen. The samples were processed through mRNA fragmentation (300 bp), followed by cDNA synthesis, library

construction using the NEB Next Ultra RNA Library Prep Kit for Illumina, PCR amplification, quality control of the library, sequencing, and subsequent analysis.

Safety assessment of Pri-MP in vivo

Mice were randomly assigned to one of four experimental groups: the Control group, the Melphalan group, the Pri-MP group, and the combination therapy group. Throughout the intervention period, body weight was monitored daily. After the treatment regimen, blood samples were collected for a comprehensive hematological analysis, including a complete blood count and assessments of hepatotoxicity and nephrotoxicity. Following euthanasia, major organs (heart, liver, spleen, lungs, and kidneys) were harvested. The tissues were then fixed, embedded, and sectioned for histological examination. Tissue sections were stained with hematoxylin and eosin (H&E) to facilitate morphological analysis under light microscopy.

Electroretinography

Intravitreal injections of various drug concentrations and combinations were administered to mice. Electroretinography (ERG) recordings were performed using a Roland Consult RETIport/scan21 system coupled with a Ganzfeld stimulator and RETIport software by an operator blinded to treatment groups. All procedures were conducted under dark-adapted conditions with minimal red-light illumination. Mice underwent overnight dark adaptation prior to testing. After anesthesia, pupils were fully dilated using mydriatic eye drops. Core body temperature was maintained at 37°C using a heated platform, and corneas were hydrated with methylcellulose solution. Bilateral recordings were acquired via gold loop corneal electrodes, with a subdermal platinum reference electrode placed on the cheek and a tail-base platinum ground electrode. Scotopic ERG responses were first evaluated with white-light flashes. Subsequently, mice were light-adapted under background illumination for 600 seconds, followed by photopic ERG recordings. a- and b-wave amplitudes and implicit times were automatically quantified by the RETIport software.

Dynamic metabolic processes and organ distribution of Pri-MP

BALB/c mice were administered an intravitreal injection of 0.4 μg of Pri-MP. The mice were subsequently euthanized at intervals of 0, 2, 6, and 12 hours and 1, 2, 3, and 7 days following the injection. The concentration of ^{197}Au in the systemic circulation was quantified *via* ICP-MS. Furthermore, various organs, including ocular structures, the heart, liver, spleen, lungs, and kidneys, were harvested to determine ^{197}Au concentrations using ICP-MS.

Statistical analysis

For normally distributed data, the mean values were reported as mean ± standard deviation (mean ± SD). Group comparisons for quantitative data were conducted using t-tests or one-way analysis of variance (ANOVA). The significance level was set at $\alpha = 0.05$, and $P < \alpha$ was considered statistically significant. *$p<0.05$, **$p<0.01$, ***$p<0.001$.
